# A higher-order numerical framework for stochastic simulation of chemical reaction systems

**DOI:** 10.1186/1752-0509-6-85

**Published:** 2012-07-15

**Authors:** Tamás Székely, Kevin Burrage, Radek Erban, Konstantinos C Zygalakis

**Affiliations:** 1 Department of Computer Science, University of Oxford, Oxford, OX1 3QD, UK; 2 Department of Mathematics, Queensland University of Technology, Brisbane, Qld 4001, Australia; 3 Mathematical Institute, University of Oxford, Oxford, OX1 3LB, UK; 4 School of Mathematics, University of Southampton, Southampton, SO17 1BJ, UK; 5 Mathematics Section, École Polytechnique Fédérale de Lausanne, Station 8, CH-1015 Lausanne, Switzerland

**Keywords:** Stochastic simulation algorithms, *τ*-leap, High-order methods, Monte Carlo error

## Abstract

**Background:**

In this paper, we present a framework for improving the accuracy of fixed-step methods for Monte Carlo simulation of discrete stochastic chemical kinetics. Stochasticity is ubiquitous in many areas of cell biology, for example in gene regulation, biochemical cascades and cell-cell interaction. However most discrete stochastic simulation techniques are slow. We apply Richardson extrapolation to the moments of three fixed-step methods, the Euler, midpoint and *θ*-trapezoidal *τ*-leap methods, to demonstrate the power of stochastic extrapolation. The extrapolation framework can increase the order of convergence of any fixed-step discrete stochastic solver and is very easy to implement; the only condition for its use is knowledge of the appropriate terms of the global error expansion of the solver in terms of its stepsize. In practical terms, a higher-order method with a larger stepsize can achieve the same level of accuracy as a lower-order method with a smaller one, potentially reducing the computational time of the system.

**Results:**

By obtaining a global error expansion for a general weak first-order method, we prove that extrapolation can increase the weak order of convergence for the moments of the Euler and the midpoint *τ*-leap methods, from one to two. This is supported by numerical simulations of several chemical systems of biological importance using the Euler, midpoint and *θ*-trapezoidal *τ*-leap methods. In almost all cases, extrapolation results in an improvement of accuracy. As in the case of ordinary and stochastic differential equations, extrapolation can be repeated to obtain even higher-order approximations.

**Conclusions:**

Extrapolation is a general framework for increasing the order of accuracy of any fixed-step stochastic solver. This enables the simulation of complicated systems in less time, allowing for more realistic biochemical problems to be solved.

## Background

Biochemical systems with small numbers of interacting components have increasingly been studied in recent years, as they are some of the most basic systems in cell biology [1-3]. Stochastic effects can strongly influence the dynamics of such systems. Applying deterministic ordinary differential equation (ODE) models to them, which approximate particle numbers as continuous concentrations, can lead to confusing results [4,5]. In some cases, even systems with large populations cannot be accurately modelled by ODEs. For instance, when close to a bifurcation regime, ODE approximations cannot reproduce the behaviour of the system for some parameter values [6]. Stochastic systems can be modelled using discrete Markov processes. The density of states of a well-stirred stochastic chemical reaction system at each point in time is given by the chemical master equation (CME) [7,8]. The stochastic simulation algorithm (SSA) [9] is an exact method for simulating trajectories of the CME as the system evolves in time.

The SSA can be computationally intensive to run for realistic problems, and alternative methods such as the *τ*-leap have been developed to improve performance [10]. Instead of simulating each reaction, the *τ*-leap performs several reactions at once, thus ‘leaping’ along the history axis of the system. This means that, unlike the SSA, the *τ*-leap is not exact; accuracy is maintained by not allowing too many reactions to occur per step. The size of each timestep, *τ*, determines the number of reactions occurring during that step, given by a Poisson random number.

This gain in speed must be balanced with loss of accuracy: larger steps mean fewer calculations but reduced accuracy. Many common *τ*-leap implementations employ a variable stepsize, as using the optimal stepsize *τ* at each point is crucial for the accuracy of the method [10-12]. However a fixed-step implementation can be useful in some cases. Although it may be less efficient, it is much easier to implement than variable-step equivalents. More importantly, the extrapolation framework that we describe in this paper requires a fixed-step method.

The original *τ*-leap as described by Gillespie [10] is known as the Euler *τ*-leap, as it can be compared to the Euler method for solving ODEs. It has been shown to have weak order of convergence one under both the scaling τ→0 (traditional scaling) [13,14] and V→∞ (large volume scaling) [15], where *V * is the volume of the system. In the same paper, Gillespie also proposed the midpoint *τ*-leap method [10], which has higher-order convergence in some cases [15,16]. Tian and Burrage [17] proposed a variant known as the Binomial *τ*-leap method that avoids issues with chemical species becoming negative. Only recently has more work been done on constructing higher-order stochastic methods. One such method is the random-corrected *τ*-leap [18], where at each timestep a random correction is added to the Poisson random number that determines the number of reactions in that step. Given a suitable random correction, the lowest order errors on the moments can be cancelled. In this way methods with up to weak order two convergence for both mean and covariance have been constructed. More recently, Anderson and Koyama [19] and Hu *et al.*[20] proposed another weak second-order method, the *θ*-trapezoidal *τ*-leap, which is an adaptation of the stochastic differential equation (SDE) solver of Anderson and Mattingly [21] for the discrete stochastic case.

In this paper we introduce a framework for improving the order of accuracy of existing fixed-step stochastic methods by using them in conjunction with Richardson extrapolation, a well-known technique for improving the order of accuracy of a numerical solver by combining sets of simulations with different stepsizes [22]. Extrapolation was originally developed for ODE solvers but has also been successfully applied to SDE methods [23]. Our approach has three main advantages: 

(1) It increases the order of accuracy of the methods supplied to it. This is desirable for the obvious reason that the resulting solutions are more accurate, as well as that larger timesteps can be used to reach a certain level of accuracy, reducing the computational cost. This is discussed further in our Conclusions.

(2) It can be applied to any fixed-step solver, for instance inherently higher-order methods such as the θ-trapezoidal τ-leap or methods with an extended stability region such as stochastic Runge-Kutta methods [24].

(3) The resulting higher-order solutions can be extrapolated again to give solutions with even higher order, as there is no (theoretical) limit on the number of times a method can be extrapolated (although statistical errors can obscure the results if the method is too accurate - see Section Monte Carlo error).

Our extrapolated methods may be useful for researchers in biology and biochemistry, as they are easy to implement and can accurately and quickly simulate discrete stochastic systems that could otherwise be too computationally intensive.

We show how the extrapolation framework can be applied to fixed-step stochastic algorithms using the examples of the fixed-step Euler *τ*-leap, midpoint *τ*-leap [10] and *θ*-trapezoidal *τ*-leap [20] methods. The extrapolation procedure depends heavily on the the existence of an appropriate global error expansion for the weak error of the numerical method. Once this is known, extrapolation consists of simple arithmetic. We calculate such an expansion for an arbitrary weak first-order method; this allows us to use extrapolation in order to obtain higher-order solutions. The weak order of all the moments of such methods can be improved by extrapolation. To reinforce this, we perform a simple error analysis by comparing the equations for the true and numerical mean of the Euler *τ*-leap method; we see that its global error is order one, and extrapolating it increases the order to two for the case of zeroth-order and first-order reactions. Using numerical simulations, we demonstrate that this is true for two first-order and three higher-order test systems with the Euler, midpoint and *θ*-trapezoidal *τ*-leap methods. Moreover, the extrapolated methods have consistently lower errors, and in many cases visibly higher-order convergence in the first two moments (the lack of convergence in some of the simulations is discussed in Section Monte Carlo error). Finally, we demonstrate that the extrapolation framework can be used to give even higher-order numerical solutions by applying a second extrapolation to the Euler *τ*-leap method.

The rest of this paper is organized as follows. We begin with an overview of the SSA and the *τ*-leap methods we will use later. We then discuss Richardson extrapolation for ODEs and SDEs and introduce the extrapolated discrete stochastic framework. We give numerical results to support our claims that extrapolation reduces the error of fixed-step methods. Finally, we discuss the Monte Carlo error and give our conclusions. The derivations of the global error expansions for SDEs and discrete stochastic methods and related material are presented in the Appendix.

### Overview of stochastic simulation methods

#### SSA

Gillespie’s SSA [9] is a statistically exact method for simulating paths from a Markov jump process. The two basic assumptions of the SSA are (i) that individual molecules are not explicitly tracked, and (ii) there are frequent non-reactive collisions. Thus we assume that the system is well-mixed and homogeneous.

The SSA simulates a system of biochemical reactions with *N* species and *M* reactions, interacting inside a fixed volume V at constant temperature. The populations of chemical species (as molecular numbers, not concentrations) at time *t* are represented as a state vector $x\equiv X(t)\equiv {\left(X_{1},\ldots ,X_{N}\right)}^{T}$. Reactions are represented by a stoichiometric matrix $\nu_{j}\equiv {\left(\nu_{1j},\ldots ,\nu_{\mathrm{Nj}}\right)}^{T}$, where *j* = 1,…,*M*, composed of *M* individual stoichiometric vectors. Each stoichiometric vector represents a reaction *j* occurring and the system changing from state **x** to **x** + _***ν****j*_. Each reaction occurs in an interval *tt* + *τ*) with relative probability *a*_*j*_(**x**)*dt*, where *a*_*j*_ is the propensity function of the *j*-th reaction. Propensity functions are given by the mass-action kinetics of the reactant chemical species. For more detail, the reader is referred to Ref. [9]. The variables **X**,***ν***_*j*_ and *a*_*j*_(**X**) fully characterise the system at each point in time.

**Algorithm 1**. SSA Direct Method *With the system in state***X**_*n*_ at time *t*_*n*_*:*

1. Generate two random numbers r1and r2from the unit-interval uniform distribution U(0,1).

2. Find the time until the next reaction $\tau =\frac{1}{a_{0}}\ln\left(\frac{1}{r_{1}}\right)$, where $a_{0}(X_{n})=\overset{M}{\underset{j=1}{\sum }}a_{j}(X_{n})$.

3. Find next reaction *j* from $\overset{j-1}{\underset{m=1}{\sum }}a_{m}(X_{n})<a_{0}r_{2}\leq \overset{M}{\underset{m=j}{\sum }}a_{m}(X_{n})$.

4. Update *t*_*n* + 1_ = *t*_*n*_ + *τ* and **X**_*n*+ 1_ = **X**_*n*_ + ***ν***_*j*_.

The Direct Method requires two newly-generated random numbers at each timestep. Although there are other SSA implementations, such as the Next Reaction Method [25] and the Optimised Direct Method [26], which can be more economical, in general the SSA is computationally costly.

#### *τ*-leap method

The *τ*-leap algorithm leaps along the history axis of the SSA by evaluating groups of reactions at once [10]. This means significantly fewer calculations, i.e. shorter computational time, per simulation, but simulation accuracy is compromised: we do not know exactly how many reactions occurred during each time step, nor can we tell more precisely when each reaction occurs than in which timestep. The *leap condition* defines an upper bound for the size of each timestep *τ*: it must be so small that the propensities do not change significantly for its duration, i.e. the change in state from time *t* to *t* + *τ* is very small [10]. Since *τ* is small, the probability *a*(**x**)*τ* that a reaction occurs during *tt* + *τ*) is also small, so the number of times *K*_*j*_each reaction fires over one timestep can be approximated by $P(a_{j}(x)\tau )$, a Poisson random variable with mean and variance *a*_*j*_(**x**)*τ*[10]. The Euler *τ*-leap algorithm is the basic *τ*-leap method, and corresponds to the Euler method for solving ODEs or the Euler-Maruyama method for solving SDEs.

**Algorithm 2**. Euler *τ*-leap method *With the system in state***X**_*n*_*at time t*_*n*_*, and a timestep τ:*

1. Generate *M* Poisson random numbers $k_{j}=P(a_{j}(X_{n})\tau )$.

2. Update *t*_*n* + 1_ = *t*_*n*_ + *τ* and ${X{}_{n}}_{+1}=X_{n}+\overset{M}{\underset{j=1}{\sum }}\nu_{j}k_{j}$.

The Euler *τ*-leap has weak order one [13-15]. Although considerable work has been done on improving the mechanism for selecting the timesteps *τ*[10-12] and eliminating steps that would result in negative populations [17,27-29], this does not affect the order of the method, limiting its accuracy. Methods with higher order are the only way to improve the accuracy beyond a certain point. Realising this, Gillespie also proposed a higher-order *τ*-leap method, the midpoint *τ*-leap [10]. This is similar to the midpoint method for ODEs, where at each step an estimate is made of the gradient of **X** at *t*_*n*_ + *τ*/2. **X**_*n*_ is then incremented using this extra information to give a more accurate approximation.

**Algorithm 3**. Midpoint *τ*-leap method *With the system in state***X**_*n*_* at time t*_*n*_*, and a timestep τ:*

1. Calculate $X^{\prime }=X_{n}+\frac{1}{2}\tau \overset{M}{\underset{j=1}{\sum }}\nu_{j}a_{j}(X_{n})$.

2. Generate M Poisson random numbers $k_{j}=P(a_{j}(X^{\prime })\tau )$.

3. Update *t*_*n* + 1_ = *t*_*n*_ + *τ* and ${X{}_{n}}_{+1}=X_{n}+\overset{M}{\underset{j=1}{\sum }}\nu_{j}k_{j}$.

Although under the scaling τ→0 the midpoint *τ*-leap has the same order of accuracy in the mean as the Euler *τ*-leap method, under the large volume scaling it has weak order two [15,16]. Our numerical simulations also suggest that it gives higher-order approximations to the first two moments for both linear and non-linear systems (although this is not clear from the literature). However the local truncation error of its covariance is first-order [16].

#### *θ*-trapezoidal *τ*-leap method

Based on the SDE method of Anderson and Mattingly [21], the *θ*-trapezoidal *τ*-leap [20] is a weak second-order method. It consists of two steps, a predictor step with size *θτ* and a corrector step with size (1 − *θ*)*τ* that aims to cancel any errors made in the first step.

**Algorithm 4**. *θ*-trapezoidal *τ*-leap method *For a specified θ *∈ (0,1), $\alpha_{1}=\frac{1}{2(1-\theta )\theta },\alpha_{2}=\frac{{\left(1-\theta \right)}^{2}+\theta^{2}}{2(1-\theta )\theta }$. *With the system in state***X**_*n*_*at timet*_*n*_*, and a timestepτ:*

1. Generate *M* Poisson random numbers $k_{j}^{\prime }=P(a_{j}(X_{n})\theta \tau ),j=1,\ldots ,M$.

2. Calculate predictor step $X^{\prime }=X_{n}+\overset{M}{\underset{j=1}{\sum }}\nu_{j}k_{j}^{\prime }$.

3. Calculate $l_{j}=\text{max}\left(\alpha_{1}a_{j}(X^{\prime })-\alpha_{2}a_{j}(X_{n}),0\right)$.

4. Generate *M* Poisson random numbers $k_{j}=P(l_{j}(1-\theta )\tau ),j=1,\ldots ,M$.

5. Update *t*_*n* + 1_ = *t*_*n*_ + *τ* and ${X{}_{n}}_{+1}=X^{\prime }+\overset{M}{\underset{j=1}{\sum }}\nu_{j}k_{j}$.

Specifically, the *θ*-trapezoidal *τ*-leap method was shown to have weak order of convergence two in the moments, and a local truncation error of $O(\tau^{3}V^{-1})$ for the covariance. *τ* = *V*^−*β*^, 0 < *β* < 1 and in the analysis V→∞, but in simulations the system volume is kept constant; thus it seems that in practice this also results in weak second-order convergence in the covariance [20].

## Methods

### The extrapolation framework

We start with an ODE solver with stepsize *h* approximating the true solution **x**(*T*) by $x_{n}^{h}$ (where *T* = *nh*), for which we assume the following global error expansion exists:

(1) $$x(T)-x_{n}^{h}=e_{k}(T)h^{k}+e_{k+1}(T)h^{k+1}+e_{k+2}(T)h^{k+2}+\ldots ,$$

where *k* is the order of the numerical method and the **e**_*k*_(*T*) are constants that only depend on the final integration time *T*. Extrapolating has the effect of cancelling the leading error term, resulting in a more accurate approximation. The existence of such an expansion is key to constructing a higher-order approximation, as the appropriate extrapolation coefficients must be used for the leading error terms to cancel. For example, in the case of a first-order method with stepsize *h*,

(2) $$x(T)-x_{n}^{h}=e_{1}(T)h+e_{2}(T)h^{2}+O(h^{3}),$$

and similarly for stepsize $\frac{h}{2}$,

(3) $$x(T)-x_{2n}^{h/2}=e_{1}(T)\frac{h}{2}+e_{2}(T)\frac{h^{2}}{4}+O(h^{3}).$$

Setting ${\hat{x}}_{n}^{h}=2x_{2n}^{h/2}-x_{n}^{h}$ and using (3) and (2), we obtain

(4) $$x(T)-{\hat{x}}_{n}^{h}=-\frac{h^{2}}{2}e_{2}(t)+O(h^{3}),$$

which implies that ${\hat{x}}_{n}^{h}$ is now a second-order approximation to **x**(*T*).

We define $z(k,q)=x_{n}^{h_{q}}$ to be a series of approximations with order *k* and stepsize *h*_*q*_to the true solution **x**(*T*), where *T* = *nh*_1_, *h*_*q*_ = *T* / *p*_*q*_ and *h*_1_ > *h*_2_ > … > *h*_*q*_. In general, one can use an order *k* method with step-sizes *hq*_−1_ and *h*_*q*_ (in the previous example, *hq*_−1_ = *h*and $h_{q}=\frac{h}{2}$, i.e. *p* = 2), to arrive at an order *k* + 1 estimate to **x**(*T*),

$$x(T)-z(k,h_{q-1})=O(h^{k+1}),$$

where

$$z(k,h_{q-1})=\frac{p^{k}\;z(k,q-1)-z(k,q)}{p^{k}-1}.$$

This process can be repeated indefinitely. We can extrapolate from the initial approximations *z*(1,1),…,*z*(1,*q*) by combining the successive solutions in each column of the Romberg table:

$$\begin{array}{cccc} z(1,1) & & & \\ & z(2,1) & & \\ z(1,2) & & z(3,1) & \\ & z(2,2) & \vdots & z(q,q)\\ z(1,3) & \vdots & z(3,q) & \\ \vdots & z(2,q) & & \\ z(1,q) & & & \end{array}$$

For instance, in Eq. (4) we used (with *p* = 2) $x_{n}^{h}=z(1,1)$ and $x_{2n}^{h/2}=z(1,2)$ to find ${\hat{x}}_{n}^{h}=z(2,1)$. Repeating with $x_{2n}^{h/2}$ and $x_{2n}^{h/4}$, we could extrapolate to find ${\hat{x}}_{2n}^{h/2}=z(2,2)$. Then we could extrapolate ${\hat{x}}_{n}^{h}$ and ${\hat{x}}_{2n}^{h/2}$ to find a third-order approximation ${\hat{\hat{x}}}_{n}^{h}=z(3,1)$, and so on.

Stochasticas

(5) $$|E(f(x(T)))-E(f(x_{n}^{h}))|,$$

where $f:R^{N}\mapsto R$ is a suitable smooth functional, for example the first moment of one of the components of **x**. $x_{n}^{h}$ is a numerical approximation to the SDE

(6) $$dX_{t}=a(X_{t},t)\mathrm{dt}+b(X_{t},t)dW_{t},$$

where $a(x,t):R^{N+1}\mapsto R^{N}$$b(x,t):R^{N+1\times M}\mapsto R^{N\times M}$ and *W*_*t*_ is a standard *M*-dimensional Wiener increment. Talay and Tubaro [23] derived a similar expansion to Eq. (1) for the global error when $x_{n}^{h}$ was calculated using the Euler-Maruyama and Milstein schemes (outlined in Appendix A). By using this expansion and the extrapolation framework, they were able to derive a second-order approximation to E(f(x(t))). The crucial step in obtaining the global error expansion was to express it as a telescopic sum of the local errors. Liu and Li [30] also followed a similar procedure to derive a global error expansion for numerical methods for SDEs with Poisson jumps, thus allowing them to obtain higher-order weak approximations.

### Extrapolation for discrete chemical kinetics

The extrapolation framework can be extended to the discrete stochastic regime. Since it requires two or more sets of approximations with given stepsizes (e.g. *h* and *h*/2), *it can only be used with a fixed-step method*: as more complex *τ*-leap methods vary *τ* at each step, it is not clear how to extrapolate them. However, this has the advantage of making our method very easy to program, as there is no complex programming overhead, for instance in choosing the timestep for *τ*-leap methods. We stress that we mostly use extrapolation to obtain higher-order approximations to the *moments* of the system (or their combinations, such as the covariance). In principle, given enough of the moments, the full probability distribution at some given time could be constructed. This is known as the Hamburger moment problem [31] and in general is a difficult problem to solve, as it might admit an infinite number of solutions. However, in some cases it is possible to reconstruct the full distribution from the extrapolated moments, as we have *a priori* knowledge about its shape. For instance, when the final distribution of states is known to be binomial, only the mean and variance are necessary for constructing the full extrapolated distribution (see Numerical Results, System 1).

In this section we focus on the Euler *τ*-leap method (ETL), since this choice simplifies the analysis, but we show in Appendix B that *any* fixed-stepsize method with known weak order can be extrapolated. In our numerical investigations we show results for the ETL, the midpoint *τ*-leap (MPTL) and the *θ*-trapezoidal *τ*-leap (TTTL) method. Extrapolating the ETL is very similar to extrapolating an ODE solver. The extrapolated ETL, which we call xETL from here on, involves running two sets of *S* ETL simulations for time *T* = *nτ*.

**Algorithm 5**. Extrapolated Euler *τ*-leap method (xETL) 

1. Run S ETL simulations with stepsize τ, to get ${}_{s}x_{n}^{\tau },s=1,\ldots ,S$.

2. Calculate desired moments $E_{S}(f(x_{n}^{\tau }))=\frac{1}{S}\overset{S}{\underset{s=1}{\sum }}f{(}_{s}x_{n}^{\tau })$.

3. Repeat steps 1 and 2 using stepsize $E_{S}(f(x_{2n}^{\tau /2}))$.

4. Take $\left(2E_{S}(f(x_{2n}^{\tau /2}))-E_{S}(f(x_{n}^{\tau }))\right)$ as the extrapolated approximation to the desired moment.

Algorithm 5 can be easily modified for use with any other fixed-step method, by replacing the ETL in Step 1 with the chosen method.

It is instructive to use a simple example to see analytically the effects of extrapolating the ETL. When the propensity functions are linear (i.e. the system only contains zeroth-order and first-order reactions), the equations for the moments are closed [32,33] and we can find explicitly the global error expansion for the first moment of our numerical solution (i.e. choose *f*(**x**) = **x**). The propensity functions can be written as

(7) $${\left[a_{1}(x),a_{2}(x),\ldots ,a_{M}(x)\right]}^{T}=Cx+d,$$

where $C\in R^{M\times N},\nu \in R^{N\times M}$ and $d\in R^{M}$, and we define *W* = *νC*, i.e. $W\in R^{N\times N}$. Thus at some timestep *mmτ* < *T*, the ETL gives (using matrix notation)

$$x_{m+1}^{\tau }=x_{m}^{\tau }+\nu P(\tau (Cx_{m}^{\tau }+d)),$$

where $x_{m}^{\tau }$ is an approximation to the true state vector **x**(*t*) at the *m*-th timestep (i.e. time *t*) with stepsize *τ*. Taking the expectation of both sides, the ETL evolves the mean as

(8) $$\begin{array}{ccc} \;E(x_{m+1}^{\tau }) & = & E(x_{m}^{\tau })+\nu E(P(\tau (Cx_{m}^{\tau }+d)))\\ & = & E(x_{m}^{\tau })+\nu E(E(P(\tau (Cx_{m}^{\tau }+d))|x_{m}^{\tau }))\\ & = & (I+\mathrm{\tau W})E(x_{m}^{\tau })+\tau \nu d, \end{array}$$

where *I* is the *N* × *N* identity matrix. Note that we cannot evaluate the expectation of $P(\tau (Cx_{m}^{\tau }+d))$ directly: because $x_{m}^{\tau }$ is a random variable, we do not know the distribution of $P(x_{m}^{\tau })$. Using the law of total expectation we can condition on $x_{m}^{\tau }$ taking a specific value; $P(x_{m}^{\tau })|x_{m}^{\tau }$ does have a Poisson distribution. From (8), we see that at time *T* = *nτ*

(9) E(xnτ)=I+τnW+n2τ2W2+…μ(0)+W−1(τnW+12τ2n2W2+…)νd.

The probability density function of the SSA at time *t* is given by the CME [7,8]. The mean μ(t)=E(x(t)) can be found from the CME [34]; it evolves as

$$\frac{d\mu (t)}{\mathrm{dt}}=W\mu \left(t\right)+\nu d.$$

The solution of this is

(10) $$\mu (t)=e^{\mathrm{Wt}}\mu (0)+e^{\mathrm{Wt}}\int_{0}^{t}e^{-\mathrm{Ws}}\nu d\;\mathrm{ds.}$$

Using a Taylor expansion and basic manipulation, at *t* = *T*this evaluates to

(11) $$\begin{array}{lll} \mu (T) & =\left(I+\mathrm{TW}+\frac{1}{2}T^{2}W^{2}+\ldots \right)\mu (0)\; & \;\\ & \;+W^{-1}\left(\mathrm{TW}+\frac{1}{2}T^{2}W^{2}+\ldots \right)\nu d.\; \end{array}$$

Taking (11) minus (9) we see that the global error is

(12) $$\begin{array}{lll} \mu (t)-E(x_{n}^{\tau }) & =\left(\frac{1}{2}\mathrm{\tau T}W^{2}+O(\tau^{2})\right)\mu (0)\; & \;\\ & \;+\left(\frac{1}{2}\mathrm{\tau TW}+O(\tau^{2})\right)\nu d.\; \end{array}$$

Furthermore the extrapolated error is

$$\begin{array}{lll} \mu (T) & \;-\;\left(2E(x_{2n}^{\tau /2})\;-\;E(x_{n}^{\tau })\right)\;=\;\left(\frac{1}{6}\tau^{2}TW^{3}\;+\;O(\tau^{3})\right)\mu (0)\; & \;\\ & \;+\left(\frac{1}{6}\tau^{2}TW^{2}+O^{3}\right)\nu d,\; & \; \end{array}$$

so the leading error term has been cancelled, leaving an order two approximation. Such a calculation would also apply for the MPTL. The difference is that for linear systems the MPTL is second-order convergent with respect to the mean [16], and similarly for the TTTL [20]. This should be taken into account in order to choose the correct extrapolation coefficients.

The above analysis only applies for the mean of a linear system, a very restricted case, but it is useful for demonstrating the basic principles of stochastic extrapolation. We employ a similar approach to Talay and Tubaro [23] and Liu and Li [30] to find a general expression for the global error expansion of the moments of a weak first-order discrete stochastic method; this is Appendix B. In Appendix C we explicitly evaluate this for the particle decay system and show that it is equivalent to Eq. 12 in this case. Appendix D contains the equations for the second moment for the case of linear systems.

#### Multimodal systems

As discussed before, one limitation of our approach is that only specific characteristics of the particle distribution can be extrapolated, rather than the full distribution. Typically we choose these to be the first and second moments, as for many systems these are the quantities of interest. However, in some cases the moments do not take values relevant to the actual dynamics of the system [35,36]. This occurs, for instance, in bimodal or multimodal systems, which have two or more stable states. Nevertheless, our method can be easily generalised to accommodate multimodal distributions as follows.

**Algorithm 6**. Extrapolated Euler *τ*-leap method (xETL) for multimodal systems 

1. Run S ETL simulations with stepsize τ, to get ${}_{s}x_{n}^{\tau },s=1,\ldots ,S$.

2. Plot histograms of the particle populations at time *T* and identify the stable states.

3. Choose point(s) at which to partition the *S* simulations into p subsets of *S*_1_,…,*S*_p_ simulations clustered around each stable state.

4. Calculate desired moments over the subsets of simulations, $E_{S_{i}}(f(x_{n}^{\tau }))=\frac{1}{S_{i}}\sum_{s=1}^{S_{i}}f{(}_{s}x_{n}^{\tau }),i=1,\ldots ,p$.

5. Repeat steps 1 and 4 using stepsize τ/2 to get $E_{S_{i}}(f(x_{2n}^{\tau /2})),i=1,\ldots ,p$.

6. Take $\left(2E_{S_{i}}(f(x_{2n}^{\tau /2}))-E_{S_{i}}(f(x_{n}^{\tau }))\right),i=1,\ldots ,p$ as the extrapolated approximation to the desired moment for each of the p subsets of simulations.

Algorithm 6 is also simple to code and does not require significant extra computational time compared to Algorithm 5 because the dynamics of the system are found from the original simulations that are necessary for the extrapolation anyway. The point(s) at which the simulations are split into subsets can affect the accuracy of the results, so must be chosen with some care. In the Numerical Results (System 5), we apply Algorithm 6 to a bimodal system, and investigate the effects of the choice of splitting point.

## Results and discussion

### Numerical results

We simulate some example systems for various stepsizes *τ* over time *t* = [0,*T*] using three fixed-step numerical methods: the Euler *τ*-leap (ETL), midpoint *τ*-leap (MPTL) and *θ*-trapezoidal *τ*-leap (TTTL) methods (with *θ* = 0.55), and their extrapolated versions, the xETL, xMPTL and xTTTL. We plot the absolute weak errors in the mean and second moment, i.e.

(13a) $$\left|E(x(T))-E_{S}(x_{n}^{\tau })|,\;|E(x(T)x^{T}(t))-E_{S}(x_{n}^{\tau }{\left(x_{n}^{\tau }\right)}^{T})\right|$$

for the ETL, MPTL and TTTL methods and

(13b) $$\begin{array}{c} |E(x(T))-2E_{S}(x_{2n}^{\tau /2})+E_{S}(x_{n}^{\tau })|,\\ \left|E(x(T)x^{T}(t))-2E_{S}(x_{2n}^{\tau /2}{\left(x_{2n}^{\tau /2}\right)}^{T})+E_{S}(x_{n}^{\tau }{\left(x_{n}^{\tau }\right)}^{T})\right| \end{array}$$

for the extrapolated methods. Here **x**(*T*) is the analytical solution at time *T* and $E_{S}(f(x_{n}^{\tau }))$ are the moments of its approximations given by *S* simulations of a fixed-step method with stepsize *τ* run for *n* steps. For the linear systems, the true solution is calculated analytically; for the non-linear systems we use the value given by 10^6^ or 10^7^ repeats of the SSA (depending on the system). The error of a weak order *α* method with stepsize *τ* is approximately *Cτ*^*α*^, where *C* is an unknown constant. To easily see the order of accuracy of our results, we plot all the errors on log-log plots. Gradients are calculated using a least squares fit through the points. The highest level of Monte Carlo error, which can be calculated for the linear systems, is marked on the appropriate plots as a straight black line. Below this level, the absolute error results are, at least in part, essentially random (see Section Monte Carlo error). We note that in all test systems, the timesteps used were all in the useful *τ*-leaping regime: Poisson counts for each reaction channel varied between tens to hundreds.

#### System 1: Particle decay system

A simple test system is a particle decay,

$$X\overset{\text{k}}{\underset{}{\to }}\emptyset ,\;k=0.1.$$

The initial particle number was *X*(0) = 10^4^ and the simulation time was *T* = 10.4. The units here, and in the systems below, are non-dimensional. This system is useful only as a test problem, but is first-order and easily tractable. The analytical mean and second moment are

$$\begin{array}{c} \;E(X(t))=X(0)e^{-\text{kT}},\\ E(X{\left(T\right)}^{2})=X(0)e^{-\text{kT}}-X(0)e^{-2\text{kT}}+{\left(X(0)\right)}^{2}e^{-2\text{kT}}. \end{array}$$

**Figure 1:**
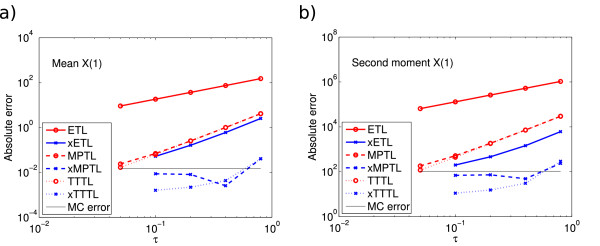
**System 1 absolute errors.** Absolute error (13) in **(a)** the mean for the ETL (gradient 1.0) and xETL (gradient 1.9), MPTL (gradient 1.9) and xMPTL (gradient 0.5), TTTL (gradient 2.0) and xTTTL (gradient 1.5); **(b)** the second moment for ETL (gradient 1.0) and xETL (gradient 1.6), MPTL (gradient 1.9) and xMPTL (gradient 0.5), TTTL (gradient 2.0) and xTTTL (gradient 1.5). The maximum Monte Carlo error levels (black straight lines) were 0.0148 (mean) and 104.8 (second moment). Results from 10^8^ simulations, each run for *T* = 10.4.

The average final particle numbers, calculated as above, were E(X(10.4))=3534.5. We ran 10^8^ simulations using timesteps *τ* = 0.05,…,0.8. The errors on the mean and second moment are shown in Figure 1. In both cases, the ETL gives first-order errors and the xETL gives approximately second-order errors. The MPTL and TTTL also converge with second order. The errors of the xMPTL and xTTTL are very small, although they do not converge with any noticeable order. This is because the values of the absolute error for these methods are effectively given by their Monte Carlo error, rather than the bias (this is a recurring theme in stochastic simulations - see Section Monte Carlo error). The maximum level of Monte Carlo error was 0.0148 for the mean and 104.8 for the second moment.

**Figure 2:**
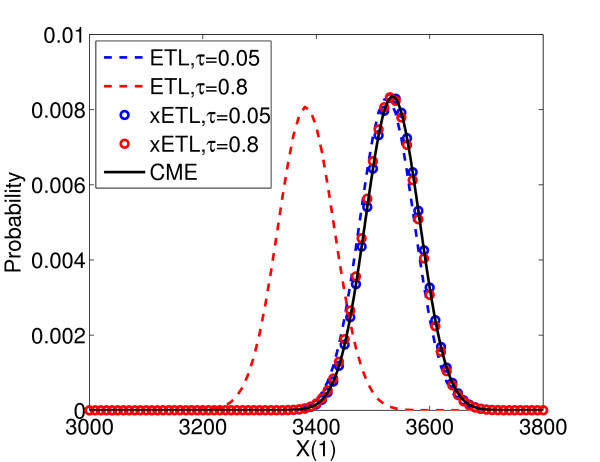
**Constructing full extrapolated distributions of System 1.** Distribution of states at time *T* = 10.4 for ETL with *τ* = 0.05 (blue dashes), ETL with *τ* = 0.8 (red dashes), xETL with *τ* = 0.05 (red circles), xETL with *τ* = 0.8 (blue circles). The analytical solution is given by the CME (black line). The extrapolated distributions match the CME solution very closely.

In addition, because the final distribution of this system is known to be binomial [10], we can construct the distribution of the extrapolated solutions from just the mean and variance (Figure 2). The dashed lines are the distributions of ETL simulations with *τ* = 0.05 (blue) and *τ* = 0.8 (red), calculated from their histograms, and the circles are the full distributions of the xETL using *τ* = 0.05 (blue) and *τ* = 0.8 (red). The solution of the CME (black line) is the true distribution. Both the extrapolated solutions match the CME solution very well, in both mean and overall shape.

#### System 2: Chain decay system

This system consists of five chemical species, each undergoing a first-order reaction. It forms a closed chain of linear reactions, and is intended as a more complicated, but still linear, example system.

$$\begin{array}{c} X_{1}\;\overset{k_{1}}{\underset{}{\to }}X_{2}\;\overset{k_{2}}{\underset{}{\to }}X_{3}\;\overset{k_{3}}{\underset{}{\to }}X_{4}\;\overset{k_{4}}{\underset{}{\to }}X_{5}\;\overset{k_{5}}{\underset{}{\to }}X_{1},\;k_{j}=0.3,\;j=1,\ldots ,5. \end{array}$$

**Figure 3:**
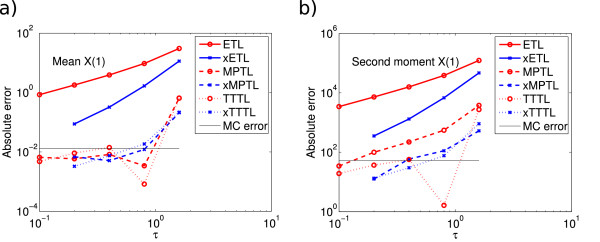
**System 2 absolute errors.** Absolute error (13) of *X*_1_ in **(a)** the mean for ETL (gradient 1.3) and xETL (gradient 2.4), MPTL (gradient 1.2) and xMPTL (gradient 1.6), TTTL (gradient 1.1) and xTTTL (gradient 1.9); **(b)** the second moment for ETL (gradient 1.3) and xETL (gradient 2.4), MPTL (gradient 1.6) and xMPTL (gradient 1.7), TTTL (gradient 1.0) and xTTTL (gradient 2.0). The maximum Monte Carlo error levels (black straight lines) were 0.0132 (mean) and 52.8 (second moment). Results from 10^8^ simulations, each run for *T* = 16.

The initial populations were **X**(0) = (2500,1875,1875,1875,1875^)*T*^and simulation time was *T* = 16. Since this system is linear, its expectation and covariance can be calculated analytically, as shown in Jahnke and Huisinga [32]; we used these to calculate the true second moment. The average final particle numbers, given by the analytical mean, were $E(X(16))={\left(1971.3,1996.7,2025.1,2020.9,1986.1\right)}^{T}$. We ran 10^8^ simulations with *τ* = 0.1,…,1.6. Figure 3 shows the absolute errors for the mean and second moment of *X*_1_. The ETL is again approximately weak order one and the xETL weak order two. Again, the errors for the MPTL, TTTL, xMPTL and xTTTL are very low, although again their order of convergence is not quite as high as expected. We believe this is due to the unusually high accuracy of these methods for closed systems compared to a maximum Monte Carlo error level of 0.0132 (mean) and 52.8 (second moment), which are high relative to the bias of these methods.

#### System 3: Michaelis-Menten system

The Michaelis-Menten is a common non-linear test system, and represents an enzyme (*X*_2_) reacting with a substrate (*X*_1_) to make a product (*X*_4_). The enzyme and substrate form a complex (*X*_3_), which can either dissociate or undergo a reaction to a product plus the original enzyme. It has four chemical species in three reactions:

$$\begin{array}{ccccc} \;X_{1} & \;+ & X_{2}\overset{k_{1}}{\to } & \;X_{3}, & \;k_{1}=10^{-5},\\ & & X_{3}\overset{k_{2}}{\to } & \;X_{1}+X_{2}, & \;k_{2}=0.2,\\ & & X_{3}\overset{k_{3}}{\to } & \;X_{2}+X_{4}, & \;k_{3}=0.2. \end{array}$$

**Figure 4:**
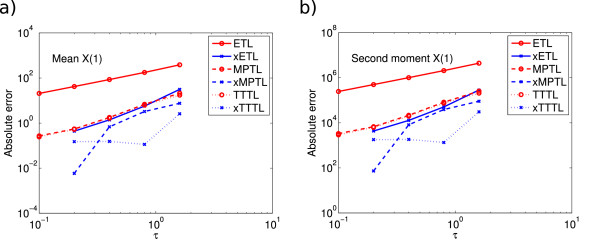
**System 3 absolute errors.** Absolute error (13) of *X*_1_ in **(a)** the mean for ETL (gradient 1.0) and xETL (gradient 2.0), MPTL (gradient 1.6) and xMPTL (gradient 3.3), TTTL (gradient 1.6) and xTTTL (gradient 1.2); **(b)** the second moment for ETL (gradient 1.0) and xETL (gradient 2.0), MPTL (gradient 1.6) and xMPTL (gradient 3.3), TTTL (gradient 1.6) and xTTTL (gradient 1.2). Results from 10^8^ simulations, each run for *T* = 16.

Simulation time was *T* = 16 and the initial populations were **X**(0) = (10^4^,2 × 10^3^,2 × 10^4^,0)^*T*^. We used 10^8^simulations with *τ* = 0.1,…,1.6. There is no analytical solution, so in this case we approximated it with 10^7^SSA simulations. The average final state, given by the SSA, was $E(X(16))={\left(5927.0,18716.2,3283.8,20789.2\right)}^{T}$. The errors in the mean and second moment for *X*_1_ are shown in Figure 4. The ETL converges with order one for 10^8^ simulations, and the xETL with order two. The MPTL and TTTL have a similar accuracy to the xETL, with approximate order two, with the xMPTL and xTTTL of approximate order three.

We investigated the effects of the coefficient of variation (CV, standard deviation divided by mean) on this system. The CVs of each species, averaged across all *τ*, in System 3 at *T* = 16 were CV(**X**(16)) = (0.01,0.003,0.02,0.004)^*T*^. In general, a higher CV indicates that the system is more noisy. We chose a new set of parameters for System 3 to give higher CVs: $X_{\text{new}}(0)={\left(100,50,200,0\right)}^{T},k_{\text{new}}={\left(10^{-4},0.05,0.07\right)}^{T}$. The CVs using these parameters were $\text{CV}(X_{\text{new}}(16))={\left(0.05,0.03,0.1,0.07\right)}^{T}$, very different from the original CVs. However the relative errors (absolute error divided by average SSA state) at *τ* = 0.1 were very similar: ${\text{error}}_{\text{rel}}(X(16))={\left(0.004,0.0005,0.003,0.001\right)}^{T}$ for the original system and ${\text{error}}_{\text{rel}}(X_{\text{new}}(16))={\left(0.001,0.0002,0.0007,0.002\right)}^{T}$ with the new parameters (note that it is not useful to average the errors across all *τ*). This shows that higher CV does not necessarily mean higher errors, and the two are indicators of different characteristics of the system. We have focused on the errors as this is the characteristic that we want to improve.

#### System 4: Two-enzyme mutual inhibition system

This is a more realistic system involving 8 chemical species and 12 reactions [37]. It represents two enzymes, *E*_*A*_ and *E*_*B*_, which catalyse the production of compounds *A* and *B*, respectively. In a classic example of double-negative feedback, each product inhibits the activity of the other enzyme. For this reason, the system is bistable in *A* and *B*: when there are many particles of *A*, few particles of *B* can be produced, and vice versa. The reactions are

$$\begin{array}{cccc} \;E_{A} & \;\overset{k_{1}}{\to } & \;E_{A}+A, & \;k_{1}=15,\\ \;E_{B} & \;\overset{k_{2}}{\to } & \;E_{B}+B, & \;k_{2}=15,\\ \;E_{A}+B & \;\overset{k_{3}}{\underset{k_{4}}{⇌}} & \;E_{A}B, & \;k_{3}=5\times 10^{-4},k_{4}=2,\\ E_{A}B+B & \;\overset{k_{5}}{\underset{k_{6}}{⇌}} & \;E_{A}B_{2}, & \;k_{5}=10^{-3},k_{6}=6,\\ \;A & \;\overset{k_{7}}{\to } & \;\emptyset & \;k_{7}=5,\\ \;E_{B}+A & \;\overset{k_{8}}{\underset{k_{9}}{⇌}} & \;E_{B}A, & \;k_{8}=5\times 10^{-4},k_{9}=2,\\ E_{B}A+A & \;\overset{k_{10}}{\underset{k_{11}}{⇌}} & \;E_{B}A_{2}, & \;k_{10}=10^{-3},k_{11}=6,\\ \;B & \;\overset{k_{12}}{\to } & \;\emptyset , & \;k_{12}=5. \end{array}$$

**Figure 5:**
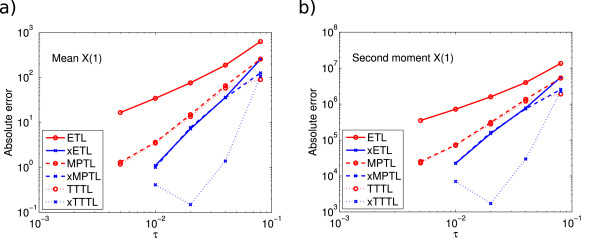
**System 4 absolute errors.** Absolute error (13) of *X*_1_ in **(a)** the mean for ETL (gradient 1.3) and xETL (gradient 2.6), MPTL (gradient 1.9) and xMPTL (gradient 2.3), TTTL (gradient 1.7) and xTTTL (gradient 2.7); **(b)** the second moment for ETL (gradient 1.3) and xETL (gradient 2.6), MPTL (gradient 2.0) and xMPTL (gradient 2.3), TTTL (gradient 1.7) and xTTTL (gradient 2.9). Results from 10^7^ simulations, each run for *T* = 3.2.

We simulated this system for *T* = 3.2 using initial populations of

$$\begin{array}{c} X(0)={\left(2\times 10^{4},1.5\times 10^{4},9500,9500,2000,500,2000,500\right)}^{T}, \end{array}$$

where $X={\left(A,B,E_{A},E_{B},E_{A}B,E_{A}B_{2},E_{B}A,E_{B}A_{2}\right)}^{T}$. We ran 10^7^ simulations of the *τ*-leap using *τ* = 0.005,…,0.08. 10^6^ SSA simulations were used as an approximation to the analytical values. The final state of the system as given by the SSA mean was E(X(3.2))=(10420.5,4884.4,3594.7,1528.0,4592.7,3812.6,3853.8,6618.2^)*T*^. The errors for *X*_1_ in this system are shown in Figure 5. The ETL and xETL gave errors of approximately order one and two, respectively. The MPTL and TTTL were again approximate order two; the xMPTL had errors very similar to those of the xETL, and the xTTTL had very low errors of approximate order three.

#### System 5: Schlögl system

**Figure 6:**
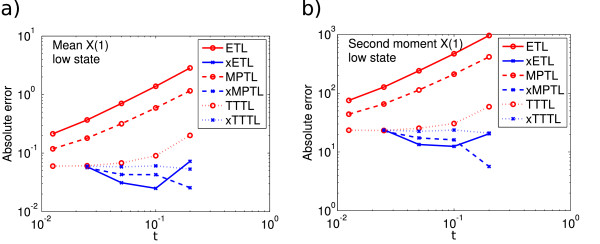
**System 5 low peak absolute errors.** Absolute error (13) of *X*_1_ in **(a)** the mean for ETL (gradient 0.9) and xETL (gradient 0.06), MPTL (gradient 0.8) and xMPTL (gradient -0.3), TTTL (gradient 0.4) and xTTTL (gradient 0.0); **(b)** the second moment for ETL (gradient 0.9) and xETL (gradient -0.1), MPTL (gradient 0.8) and xMPTL (gradient -0.6), TTTL (gradient 0.3) and xTTTL (gradient 0.0). Results from 10^8^ simulations, each run for *T* = 5.

**Figure 7:**
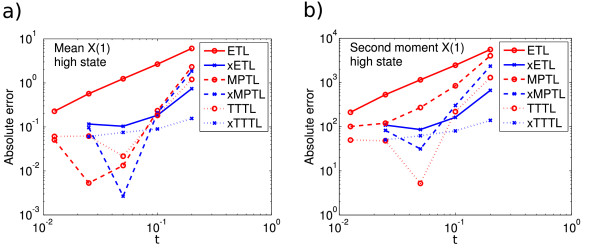
**System 5 high peak absolute errors.** Absolute error (13) of *X*_1_ in **(a)** the mean for ETL (gradient 1.2) and xETL (gradient 0.9), MPTL (gradient 1.6) and xMPTL (gradient 1.9), TTTL (gradient 1.0) and xTTTL (gradient 0.4); **(b)** the second moment for ETL (gradient 1.2) and xETL (gradient 0.9), MPTL (gradient 1.3) and xMPTL (gradient 1.8), TTTL (gradient 1.2) and xTTTL (gradient 0.5). Results from 10^8^ simulations, each run for *T* = 5.

The last example we give illustrates how our method could work for systems that have multimodal distributions. The Schlögl system consists of four reactions [38] and is bimodal:

$$\begin{array}{ccc} A+2X\overset{k_{1}}{\to } & \;3X, & \;k_{1}=3\times 10^{-7},\\ \;3X\overset{k_{2}}{\to } & \;A+2X, & \;k_{2}=10^{-4},\\ \;B\overset{k_{3}}{\to } & \;X, & \;k_{3}=10^{-3},\\ \;X\overset{k_{4}}{\to } & \;B, & \;k_{4}=3.5, \end{array}$$

where the populations of species *A* and *B* are held constant at 10^5^ and 2 × 10^5^ respectively, so only the numbers of *X* can change. This is a common benchmark system for computational simulation algorithms. Under certain parameter configurations, this system has two stable states for *X*, one high and one low. When *X*(0) is low, the system usually settles in the lower equilibrium, and vice versa for high *X*(0). We used *X*(0) = 250, an intermediate value that gives a bimodal distribution. We ran 10^8^ simulations until *T* = 5 using *τ* = 0.0125,…,0.2. Because of the bimodality, we separated the data into two sets. As the Schlögl system is simple enough to be solved using a CME solver (see e.g. [39]), we could calculate the density of states of *X* at *T* = 5. When simulating more complicated systems, this can be approximated by a histogram of the distribution of species at *T* (from the initial ETL simulations, for instance) to get a reasonable idea of its shape. Average final particle number was calculated from the CME to be E(X(5))=101.3 for the low peak and E(X(5))=546.2 for the high peak. Figures 6 and 7 show the absolute errors for this system (low and high peaks, respectively). The error levels seem to be similar in both cases. The general trend was for the ETL, MPTL and TTTL to converge with approximate order one, whereas the extrapolated solutions did not have a clear order of convergence. It is clear that in this case also, the Monte Carlo error is interfering with our ability to see a clear order of convergence.

The gradient of the MPTL mean error seems to change from approximately one (Figure 6) to around 1.5 (Figure 7). It is unlikely that this is due to Monte Carlo error, as the error of the MPTL is high enough that this should not be an issue. In fact, this is probably due to the large volume limit behaviour of the MPTL, discussed after Algorithm 3. Because the mean of the high peak is several times higher than the mean of the low peak, the system is closer to the large volume limit and the weak order of the MPTL increases accordingly. Once in the large volume limit, the gradient is expected to be two.

**Figure 8:**
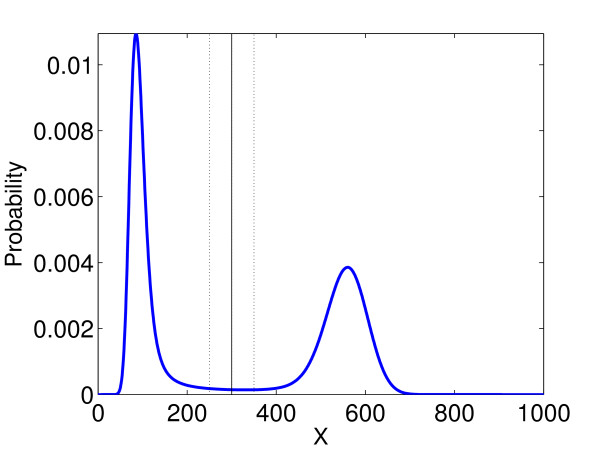
**System 5 full distribution, *T* = 5.** The distribution of System 5 calculated using a CME solver for *T* = 5. The three splitting points we have compared in this paper are illustrated.

The point at which the data is separated must be chosen carefully, as it can influence the error results. Figure 8 shows the distribution of *X* at *T* = 5 calculated using a CME solver. The three choices of splitting points that we compare have also been marked. We chose *X* = 300; this is a fairly obvious splitting point in our case. To support this, we tested the effects of splitting the data given by the ETL at *X* = 250 and *X* = 350. We calculated the relative change in mean, second moment and variance between these two splitting values, averaged over all simulations of the ETL and all five timesteps: Table 1 shows that in this case the percentage difference is relatively small for the mean, becomes larger for the second moment, and is very high for the variance. This implies that the choice of splitting point is very important in this case, and should be carefully considered.

**Table 1:** Relative differences in moments for different splitting values of Schlögl system

**Moment**	**Low peak**	**High peak**
Mean	6.4%	1.6%
Second moment	21.8%	2.5%
Variance	81.6%	50.5%

Relative differences in moments E(f(X)) for data split at *X* = 250 and *X* = 350 for the Schlögl system as percentages of their values when split at *X* = 300, i.e. $100*|E{\left(f(X)\right)}_{\text{split}350}-E{\left(f(X)\right)}_{\text{split}250}|/E{\left(f(X)\right)}_{\text{split}300}$.

### Higher extrapolation

**Figure 9:**
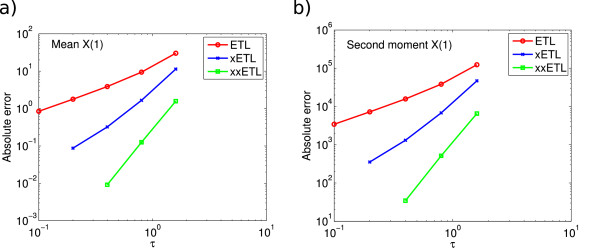
**System 2, double-extrapolated absolute errors.** Absolute error of *X*_1_ for the Euler *τ*-leap (ETL), extrapolated Euler *τ*-leap (xETL) and double-extrapolated Euler *τ*-leap (xxETL) algorithm for **(a)** the mean, gradients 1.3 (ETL), 2.3 (xETL), 3.7 (xxETL), and **(b)** the second moment, gradients 1.3 (ETL), 2.4 (xETL), 3.8 (xxETL). Results from 10^8^ simulations. Double-extrapolation produces consistently higher-order results.

**Figure 10:**
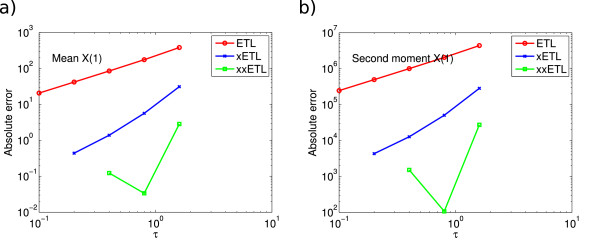
**System 3, double-extrapolated absolute errors.** Absolute error of *X*_1_ for ETL, xETL and xxETL for **(a)** the mean, gradients 1.0 (ETL), 2.0 (xETL) and 2.3 (xxETL), and **(b)** the second moment, gradients 1.0 (ETL), 2.0 (xETL), 2.1 (xxETL). Results from 10^8^ simulations. In this case, the xxETL errors do not show a clear order of accuracy, but are nonetheless smaller compared to the other methods.

In principle it is possible to use extrapolation in conjunction with some fixed-step method such as the ETL to obtain increasingly higher-order approximations using the appropriate extrapolation coefficients from the Romberg table. In practice, the usefulness of repeated extrapolations is debatable, as each adds extra computational overhead and the higher accuracy can be obscured by Monte Carlo fluctuations. However it might be useful to create up to third or fourth-order methods in this way. We tried double-extrapolating the ETL on our test systems, with reasonable success. Figure 9 shows the ETL, xETL and double-extrapolated Euler *τ*-leap (xxETL) errors on species *X*_1_ of System 2; in this case we can see that the xxETL results are approximately order three (or higher). Double-extrapolating gave substantially lower errors in almost every system, but it was not always easy to determine the order of convergence. A good example of this is System 3 in Figure 10. In such systems with relatively high Monte Carlo error, the approximate solutions from the xxETL were obscured by these fluctuations. The order of accuracy of the xxETL could be successfully seen for most molecular species of System 2, but it was not possible for the other systems as this would require a significant increase in the number of simulations, in order to further reduce the Monte Carlo error.

### Monte Carlo error

The weak error we calculate numerically is

(14) $$|E(f(x(t)))-E_{S}(f(x_{n}^{\tau }))|.$$

This error can be separated into two parts

(15) $$\left[E(f(x(t)))-E_{\infty }(f(x_{n}^{\tau }))\right]+\left[E_{\infty }(f(x_{n}^{\tau }))-E_{S}(f(x_{n}^{\tau }))\right],$$

where $E_{\infty }(f(x_{n}^{\tau }))$ are the theoretical values of the moments calculated by an infinite number of simulations with stepsize *τ*. The first term is the truncation error of the moments from their analytical solutions, i.e. the bias of the method, which depends only on the choice of timestep. The second term is the Monte Carlo error, which depends only on the number of simulations and is given by $\frac{C}{\sqrt{S}}$, where *C* is some constant and *S* the number of simulations. The Monte Carlo error can be so large that it overwhelms the bias of the underlying numerical method completely; in this case all of the numerical results are, in effect, incorrect, as they are random fluctuations.

This formulation is useful when the propensity functions are linear. In this case, the moment equations are closed, so $E_{\infty }(f(x_{n}^{\tau }))$ can be calculated for the appropriate numerical method. As an example, consider the mean of the ETL: its true value is given by Eq. (10) and the value of its numerical approximation can be found by iterating Eq. (8). In addition, a similar calculation can be found in Appendix D for the second moment. Unfortunately, this is not possible for non-linear systems, since in this case the equations describing the evolution of the moments are not closed any more [40].

**Figure 11:**
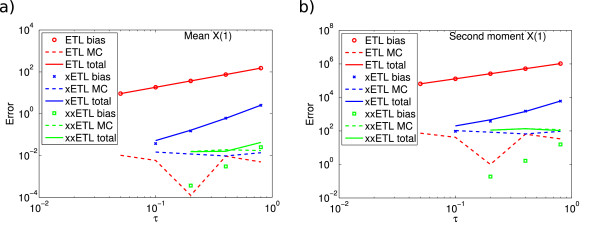
**Split errors of System 1 using ETL.** Split errors for System 1 for **(a)** the mean and **(b)** the second moment. The bias can be easily seen with the Euler *τ*-leap (ETL) or only a single extrapolation (xETL), but is obscured when we extrapolate a second time (xxETL). Results are from 10^8^ simulations.

Such a breakdown of the errors of System 1 with the ETL method is shown in Figure 11, using results from 10^8^ simulations. We see that for the case of the ETL and xETL, the bias can easily be seen as the Monte Carlo errors are relatively low compared to the bias. However, when we extrapolate a second time, the bias of the resulting estimator is so low that Monte Carlo fluctuations completely obscure it, even with 10^8^ simulations. This gives a poor approximation for the total error. The only way to reduce Monte Carlo error is to run more simulations or use variance reduction methods. This is a good illustration of why it is important to perform a suitable number of simulations to get accurate estimates of the moments.

Variance reduction methods, which aim to decrease the Monte Carlo error, are another useful way of reducing computational time: because the Monte Carlo error is lower, less simulations need to be run for a given accuracy, saving time. This is an important topic in its own right and we do not address it in this paper; we refer the interested reader to e.g. [41]. It is an active research area: recently Anderson and Higham [42] were able to significantly reduce the overal computational cost associated with the stochastic simulation of chemical kinetics, by extending the idea of multi level Monte Carlo for SDEs [43,44].

## Conclusions

Throughout this paper, we have given reduced computational time as a motivation for using the extrapolation framework. As this is an important issue, we support here our claim that extrapolation speeds up simulations at a given error level. Although twice as many simulations must be run for a single extrapolation, the loss in computational time from more simulations is compensated for by the significant reduction in error. This is important, as slow runtime is often a limitation of stochastic methods. Total computational times and the corresponding errors in the mean (in brackets) of all three methods used in this paper and their extrapolated and double-extrapolated versions are shown in Tables 2 and 3 for Systems 1 and 4 (10^8^ and 10^7^ simulations, respectively). The estimated time to run the same number of SSA simulations is given for comparison. It should be noted that all the extrapolated and double-extrapolated *τ*-leap times are estimates: the time-consuming part of the extrapolation method is the two (or more) sets of simulations of the original method that must be run; the extrapolation itself is a fast arithmetic operation. The times for a single extrapolation are calculated as *runtime*(*τ* = *h*) + *runtime*(*τ* = *h*/2), and for a double-extrapolation as *runtime*(*τ* = *h*) + *runtime*(*τ* = *h*/2) + *runtime*(*τ*=*h*/4). The extrapolated methods take several times longer to run, but the errors they give are several to hundreds of times lower. Most of the exceptions are where the error has clearly reached the Monte Carlo level (Tables 2 and 3, entries marked (-MC)). Besides extrapolation, the other obvious way to reduce error is to use a smaller timestep, so the real test for the effectiveness of extrapolation is to compare the runtimes of cases with similar error values. In Tables 2 and 3, we have marked in bold the extrapolated errors that can be directly compared to the base method (i.e. read up one row) and take less time to run, and similarly for double-extrapolated errors as compared to single-extrapolated ones. Although this varies for each system and simulation method, the general trend is that extrapolation takes less time to give a similar level of error, and this pattern was similar for our other example systems. Thus we feel that in general, extrapolation is a worthwhile procedure.

**Table 2:** Processing times of System 1

			**τ**		
	**0.05**	**0.1**	**0.2**	**0.4**	**0.8**
ETL	21.3 (9.2)	13.3 (18.4)	7.63 (37.1)	4.08 (74.7)	2.21 (152.0)
xETL	-	34.3 (0.05)	21 (0.16)	11.8 (0.61)	**6.34 (2.6)**
xxETL	-	-	(-MC) 42.1 (0.015)	(-MC) 25 (0.016)	**14 (0.043)**
MPTL	21.3 (0.024)	13.4 (0.070)	7.65 (0.25)	4.18 (1.0)	2.24 (4.2)
xMPTL	-	(-MC) 34.7 (0.0088)	(-MC) 21.1 (0.0082)	(-MC) 11.8 (0.0026)	**6.38 (0.041)**
xxMPTL	-	-	(-MC) 42.4 (0.0089)	(-MC) 25.2 (0.0090)	(-MC) 14 (0.0088)
TTTL	40.8 (0.017)	21.1 (0.063)	13.3 (0.26)	7.65 (1.1)	4.17 (4.2)
xTTTL	-	(-MC) 62.1 (0.0016)	(-MC) 34.6 (0.0022)	(-MC) 20.9 (0.0044)	**11.8 (0.041)**
xxTTTL	-	-	(-MC) 75.4 (0.0022)	(-MC) 42.2 (0.0031)	(-MC) 25.1 (0.011)

Processing times (in thousands of seconds) of 10^8^ simulations of System 1 using Euler *τ*-leap, midpoint *τ*-leap and *θ*-trapezoidal *τ*-leap methods and their extrapolated and double-extrapolated versions. Absolute errors in the mean from the analytical values are included in brackets. For comparison, implementation of 10^8^ simulations of an SSA using the Direct Method would take approximately 1.2 × 10^5^seconds. Entries marked (-MC) are thought to be below the Monte Carlo error level; bold entries are faster than the un/single-extrapolated versions with similar error level (i.e. compare with one row above).

**Table 3:** Processing times of System 4

			**τ**		
	**0.005**	**0.010**	**0.020**	**0.040**	**0.08**
ETL	191 (16.7)	82.3 (34.5)	46 (76.1)	32.6 (188.5)	19.7 (630.1)
xETL	-	293 (1.1)	**123 (7.2)**	**66.4 (36.3)**	35.1 (253.1)
xxETL	-	-	**279 (0.93)**	152 (2.5)	80.1 (36.0)
MPTL	165 (1.3)	84 (3.6)	44.9 (15.0)	24.1 (65.5)	12.3 (257.1)
xMPTL	-	244 (1.0)	129 (7.7)	69.1 (35.5)	36.3 (126.2)
xxMPTL	-	-	(-MC) 289 (1.2)	(-MC) 153 (1.5)	**81.3 (5.3)**
TTTL	277 (1.2)	155 (3.5)	82.4 (13.5)	44.6 (58.1)	23.3 (90.0)
xTTTL	-	(-MC) 430 (0.41)	(-MC) 239 (0.15)	**128 (1.4)**	68 (107.5)
xxTTTL	-	-	(-MC) 515 (0.45)	(-MC) 283 (0.37)	(-MC) 151 (16.9)

Processing times (in thousands of seconds) of 10^7^ simulations of System 4 using Euler *τ*-leap, midpoint *τ*-leap and *??*-trapezoidal *τ*-leap methods and their extrapolated and double-extrapolated versions. Absolute errors in the mean of *X*_1_from the SSA values are included in brackets. Running 10^7^ simulations of an SSA using the Direct Method would take approximately 1.47 × 10^7^ seconds. Entries marked (-MC) are thought to be below the Monte Carlo error level; bold entries are faster than the un/single-extrapolated versions with similar error level (i.e. compare with one row above).

Monte Carlo error is an unavoidable problem when using stochastic simulations. The statistical fluctuations inherent in stochastic systems can obscure the bias error (i.e. order of convergence) of the numerical method if their size relative to the bias is large, as the total error is made up of these two contributions. A large number *S* of simulations must be run, as the Monte Carlo error scales as $1/\sqrt{S}$. This error varies for each system. Figures 9 and 10 (and 11) show this clearly: both of the xxETLs have total errors of similar size for the same *τ*, but System 2 has relatively low Monte Carlo error, allowing us to see the bias of that system. However, we believe that System 3 has relatively high Monte Carlo error compared to its bias, implying that the xxETL errors we see in the figure are all due to statistical fluctuations. It should be noted that this seems to happen for all five test problems we use. The reason for this is that the extrapolated methods (and even the MPTL and TTTL, in some cases) have very high accuracy (i.e. low bias error). Since it is only possible to run a limited number of simulations, when the bias is very small, the total error will be given almost completely by the contribution from the Monte Carlo error.

A contrasting approach to reducing numerical errors is the multilevel Monte Carlo method. Originally developed for SDEs [43,44], it has recently been extended to discrete chemical kinetics [42]. By considering a formulation of the total error similar to Eq. (15), the multilevel Monte-Carlo method aims to reduce it by decreasing the Monte Carlo error. Here also many approximate solutions are generated with a variety of different timesteps. By intelligently combining many coarse-grained simulations with few fine-grained ones, it is possible to find a similar level of accuracy to just using fine-grained simulations. In contrast, extrapolation uses the same number of coarse and fine-scale solutions and gives results which are more accurate than the fine-scale solution, by reducing the bias instead of the Monte Carlo error. In cases where the bias is obscured by statistical errors, using a combination of both extrapolation and the multilevel Monte Carlo method would be ideal, as it would reduce both sources of error. This is an interesting research question and we plan to address it in the future.

In this work, we have extended the extrapolation framework, which can increase the weak order of accuracy of existing numerical methods, to the discrete stochastic regime. To demonstrate the concept, we have applied it to three fixed-step methods, the Euler, midpoint and *θ*-trapezoidal *τ*-leap methods. Thus we have demonstrated numerically the effectiveness of extrapolation on a range of discrete stochastic numerical methods with different orders of accuracy for a variety of problems. The main requirement to use extrapolation with a numerical method is the existence of an expression for the global error that relates the error to the stepsize of the method. Analytically, this is all that must be found to show higher weak order convergence of the extrapolated method. To extrapolate once, only the leading error term need be known; further extrapolation requires knowledge of higher terms. We have found the form of the global weak error for a general weak order one method; the procedure is similar for higher-order methods. This is the real power of our approach: it can be applied to *any* fixed-step numerical method. Moreover, further extrapolations can raise the order of accuracy of the method indefinitely (although beyond a certain point the lower errors will be overtaken by Monte Carlo errors). We expect our method to be useful for more complex biochemical systems, for instance where frequent reactions must be simulated fast but accuracy is still important.

## Appendices

## SDE extrapolation

We summarise below the work of Talay and Tubaro [23] on extrapolating SDEs. The global weak error of a stochastic numerical scheme is

(16) $$\text{error}(T,h)=E(f(x(t)))-E(f(x_{n}^{h})).$$

To extrapolate this scheme, we must obtain an expression of the form (1). We start with the Kolmogorov backward equations,

$$\begin{array}{lll} \;u_{t}+ℒu= & 0,\; & \;\\ \;u(x,t)= & f(x),\; & \; \end{array}$$

where $u_{t}=\frac{\mathrm{\partial u}}{\mathrm{\partial t}}$*f * is a homogeneous function on $[0,T]\times R^{N}\to R$ and u(x,t)=E(f(x(t))|x(t)=x). **x** are the solutions of the SDE (6) with initial conditions **x**(0) = **x**_0_ and ℒ is the generator of (6),

$$ℒ\equiv a(x,t)\cdot \nabla_{x}+\frac{1}{2}b(x,t)b^{T}(x,t):\nabla_{x}\nabla_{x},$$

with $A:B=\underset{i,j}{\sum }A_{\mathrm{ij}}B_{\mathrm{ij}}$. Crucially, (16) can then be written as

$$\text{error}(T,h)=Eu(x_{0},0)-Eu(x_{n}^{h},T).$$

The first term is known; the second can be found by recursively calculating the error between ***u*** when it is evaluated from adjacent timesteps:

$$Eu(x_{n}^{h},T),=Eu(x_{0},0)+h^{2}\overset{n-1}{\underset{k=0}{\sum }}E\psi (x_{k}^{h},\mathrm{kh})+h^{2}K_{n}^{h},$$

where *ψ*(**x***t*) is an error function that is well-behaved and specific to each numerical method and $K_{n}^{h}$ is a constant of unit order. For the case of the Euler-Maruyama and Milstein methods, the second term is O(h), so their global weak error is [23]

(17) $$\text{error}(T,h)=-h\int_{0}^{T}\psi E(x(s),t)\mathrm{ds}+O(h^{2}).$$

Eq. (17) shows that the Euler-Maruyama and Milstein methods have global weak order one. It is easy to see that extrapolating them leads to solutions with global weak order two.

## Discrete stochastic global error expansion

Here we derive a global error expansion similar to Eqs. (1) and (17) for the numerical approximation of moments by a weak first-order discrete stochastic method, such as the Euler or midpoint *τ*-leap methods. Once the form of the error expansion is known, it is clear what extrapolation coefficients to use and extrapolation is simple. Similarly to the case of SDEs, we start with the Kolmogorov backward equations

(18a) $$\begin{array}{cc} \partial_{t}u+ℒu=0\; & \;\text{in}\;Z_{+}^{N}\times [0,T), \end{array}$$

(18b) $$\begin{array}{cc} \;u(x,t)=f(x) & \;\text{on}\;Z_{+}^{N}\times \{T\}. \end{array}$$

Here

$$\mathrm{ℒu}\equiv \overset{M}{\underset{j=1}{\sum }}a_{j}\left(x\right)\left(u(x+\nu_{j})-u(x)\right)$$

and

$$u(x,t),=E\left(f(X_{T})|X_{t}=x\right),$$

with **X**_*t*_ = **X**(*t*). Using semigroup notation it is possible to formally denote the solution of (18) as

$$u(x,t),=e^{(T-t)ℒ}f(x),$$

which can be useful for quantifying the local error of a numerical approximation. By applying a stochastic Taylor expansion [45] to jump processes [16], the one-step expansion for the expectation of *f * calculated with a first-order numerical method should satisfy

(19) $$\begin{array}{lll} E(f(X_{h}^{h})|X_{0}^{h}=x) & =A_{h}f(x)\; & \;\\ & =f(x)+h\mathrm{ℒf}(x)+\overset{\infty }{\underset{j=2}{\sum }}\frac{h^{j}}{j!}A_{j}f(x),\; \end{array}$$

where $A_{j}$ are difference operators associated with the numerical method in hand. Furthermore we have for the true one-step expansion

(20) $$\begin{array}{lll} E(f(X_{h})|X_{0}^{h}=x) & =e^{\mathrm{ℒh}}f(x)\; & \;\\ & =f(x)+h\mathrm{ℒf}(x)+\overset{\infty }{\underset{j=2}{\sum }}\frac{h^{j}}{j!}{ℒ}^{j}f(x).\; \end{array}$$

### Remark 1

An important element in our derivation of a global error expansion relates to the boundedness of *u*(**x***t*), and its discrete derivative. This boundedess is guaranteed [14] when the number of molecules in the chemical system is conserved or decreases with time. Proving this in the general case where zeroth-order reactions can add molecules to the system is a non-trivial task. One way around this problem is to set the propensity functions *a*_*j*_(**x**) to zero outside a large but finite domain [14]; this is the approach we follow here.

**Figure 12:**
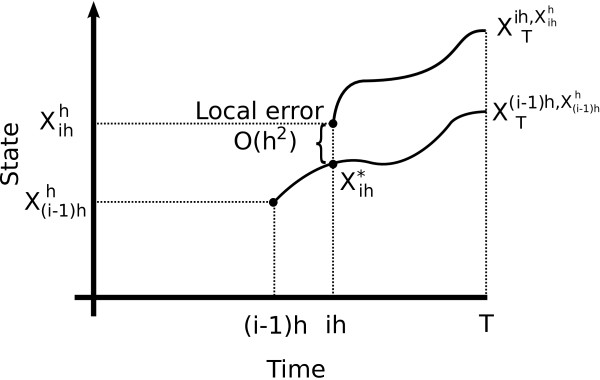
**Local error.** Representation of the local error in terms of the function $u(x,t),=E(f(X_{T}^{t,x}))$.

Now if we let *e*(*f*,*T*,*h*) define the global error in the numerical approximation of $E(f(X_{T}))$ at time *T* = *nh*by a first-order numerical method with timestep *h* we see that

$$\begin{array}{lll} e(f,T,h) & =E(f(X_{T})|X_{0}=x)-E(f(X_{T}^{h})|X_{0}^{h}=x)\; & \;\\ & =u(x,0)-E(u(X_{T}^{h},t),|X_{0}^{h}=x)\; & \;\\ & =\overset{N}{\underset{i=1}{\sum }}E(u(X_{(i-1)h}^{h},(i-1)h))-E(u(X_{\mathrm{ih}}^{h},\mathrm{ih}))\; & \;\\ & =\overset{N}{\underset{i=1}{\sum }}E(u(X_{\mathrm{ih}}^{*},\mathrm{ih}))-E(u(X_{\mathrm{ih}}^{h},\mathrm{ih})),\; & \; \end{array}$$

where we have used the result in Figure 12, and for notational simplicity omitted the dependence of the expectations on the initial conditions. If we take $g_{i}(y)=u(y,\mathrm{ih})=e^{ℒ(T-\mathrm{ih})}f(y)$, then

$$E(u(X_{\mathrm{ih}}^{*},\mathrm{ih}))-E(u(X_{\mathrm{ih}}^{h},\mathrm{ih}))=E(g_{i}(X_{\mathrm{ih}}^{*}))-E(g_{i}(X_{\mathrm{ih}}^{h})).$$

Applying Eqs. (19) and (20) to *g*_*i*_,

$$\begin{array}{cc} \begin{array}{ccc} e(f,T,h) & = & \overset{N}{\underset{i=1}{\sum }}E\left[\frac{h^{2}}{2}({ℒ}^{2}-A_{2})g_{i}(X_{(i-1)h}^{h})+h^{3}R_{i}^{h}\right]\\ & = & \overset{N}{\underset{i=1}{\sum }}E\left[\frac{h^{2}}{2}({ℒ}^{2}-A_{2})e^{-hℒ}g_{i-1}(X_{(i-1)h}^{h})\right]\\ & & +h^{3}E(R_{i}^{h})\\ & = & \overset{N}{\underset{i=1}{\sum }}\frac{h^{2}}{2}E\left[\left({ℒ}^{2}-A_{2})e^{-hℒ}u(X_{\left(i-1)h\right)}^{h},(i-1)h\right)\right]\\ & & +h^{3}E(R_{i}^{h}), \end{array} & \; \end{array}$$

where we have used the fact that $g_{i}=e^{-hℒ}g_{i-1}$. We thus obtain

(21) $$e(f,T,h)=\overset{N}{\underset{i=1}{\sum }}h^{2}E(\psi_{e}((X_{(i-1)h}^{h},(i-1)h)))+h^{3}E({\tilde{R}}_{i}^{h}),$$

where

(22) $$\psi_{e}(x,t),=\frac{1}{2}\left({ℒ}^{2}-A_{2}\right)u(x,t),,$$

and

$$E({\tilde{R}}_{i}^{h})\leq C(t),,$$

from our assumptions on the boundedness of *u*(**x**,*t*). Furthemore it is easy to see that

$$\overset{N}{\underset{i=1}{\sum }}hE\left|\psi_{e}\left((X_{(i-1)h}^{h},\left(i-1\right)h\right)\right|\leq C\left(T\right).$$

Using this and results from Talay and Tubaro [23] we find that

$$e(f,T,h)=-h\int_{0}^{T}E(\psi_{e}(X_{s},s))\mathrm{ds}+O(h^{2}),$$

where*ψ*_*e*_(**x***t*) is dependent on the numerical method and given by (22).

## An example explicit calculation of the global error expansion for a linear system

In Eq. (12) we calculated the global error on the mean for a linear system using the equations for its true and numerical solutions. We show how this approach connects to the general formulation of the errors given by Eq. (21) by using System 1 as an example. We choose this example as it is one-dimensional and linear and is simple enough that the global error Eq. (12) can be calculated explicitly from the general formulation. We define the following notation for the discrete difference:

$$f^{\nu }=f(x+\nu )-f(x),$$

where we have assumed that we have only one chemical species subject to only one chemical reaction. For this particular case,

$${ℒ}^{2}f=a^{2}f^{\nu \nu }+a^{2}a^{\nu }f^{\nu \nu }+aa^{\nu }f^{\nu },$$

while for the ETL

$$A_{2}f=a^{2}f^{\nu \nu },$$

and thus

(23) $$\begin{array}{ccc} \;\psi_{e}(x,t) & \;= & \frac{1}{2}(a^{2}(x)(a(x+\nu )-a(x))(u(x+2\nu ,t)\\ & & -2u(x+\nu ,t)+u(x,t))\\ & & +\frac{1}{2}a(x)(a(x+\nu )\;-\;a(x))(u(x+\nu ,t)\;-\;u(x,t)). \end{array}$$

For particle decay, *ν* = −1 and *a*(*x*) = *κx*. For *g*(*x*) = *x*, the solution to (18) is

$$u\left(x,t\right)=xe^{-\kappa (T-t)},$$

so (23) becomes

$$\psi_{e}\left(x,t\right)=\frac{1}{2}\kappa^{2}xe^{-\kappa (T-t)}.$$

Thus the global error given by the general formulation is

$$\begin{array}{lll} & \overset{N}{\underset{i=1}{\sum }}h^{2}E(\psi_{e}((X_{(i-1)h}^{h},(i-1)h)))\; & \;\\ & \;=\frac{\kappa^{2}h^{2}}{2}\overset{N}{\underset{i=1}{\sum }}E(X_{\left(i-1)h\right)}^{h})e^{-\kappa (T-(i-1)h)}\; & \;\\ & \;=\frac{\kappa^{2}h^{2}}{2}\overset{N}{\underset{i=1}{\sum }}{\left[(1-\mathrm{\kappa h})e^{\mathrm{\kappa h}}\right]}^{i-1}e^{-\mathrm{\kappa T}}E(X_{0}^{h})\; & \;\\ & \;=\frac{\kappa^{2}\mathrm{Th}}{2}\left(\frac{{\left[(1-\mathrm{\kappa h})e^{\mathrm{\kappa h}}\right]}^{N}-1}{(1-\mathrm{\kappa h})e^{\mathrm{\kappa h}}-1}\right)\frac{e^{-\mathrm{\kappa T}}}{N}E(X_{0}^{h})\; & \;\\ & \;=\frac{\kappa^{2}\mathrm{Th}}{2}E(X_{0}^{h})+O(h^{2}),\; & \; \end{array}$$

which agrees exactly with what one obtains by calculating Eq. (12) for System 1 (i.e. setting *d*=0,*W*=−*κ*).

## Formulae for the second moment of the Euler *τ*-leap in the case of linear systems

We can write the formulae for the numerical and analytical evaluation of the second moment of the ETL. This is useful for finding the relative contributions of the bias and Monte Carlo errors, as well as for explicitly calculating the local errors. The ETL evolves the second moment in time as

(24) $$\begin{array}{lll} E\left(x_{m+1}^{\tau },{\left(x_{m+1}^{\tau }\right)}^{T}\right)\; & =\;E\left[\;\left(x_{m}^{\tau }\;+\;\nu P(\tau (Cx_{m}^{\tau }\;+\;d))\right)\right)\; & \;\\ & \left(\;{\left(x_{m}^{\tau }\;+\;\nu P(\tau (Cx_{m}^{\tau }\;+\;d))\right)}^{T}\right]\;.\; \end{array}$$

Using the law of total expectation and writing $S_{m}^{\tau }=E(x_{m}^{\tau }{\left(x_{m}^{\tau }\right)}^{T})$, $B_{m}^{\tau }=\text{diag}(CE(x_{m}^{\tau })))$ and ***D***=diag(**d**), we obtain

(25) $$\begin{array}{c} S_{m+1}^{\tau }\;=\;S_{m}^{\tau }\;+\;\mathrm{\tau W}S_{m}^{\tau }\;+\;\tau S_{m}^{\tau }W^{T}\;+\;\tau \mu_{m}^{\tau }d^{T}\nu^{T}\;+\;\tau \nu d{\left(\mu_{m}^{\tau }\right)}^{T}\\ \;+\;\tau^{2}WS_{m}^{\tau }W^{T}\;+\;\tau^{2}W\mu_{m}^{\tau }d^{T}\nu^{T}\;+\;\tau^{2}\nu d{\left(\mu_{m}^{\tau }\right)}^{T}W^{T}\\ \;+\;\tau^{2}\nu dd^{T}\nu^{T}\;+\;\tau \nu B_{m}^{\tau }\nu^{T}+\mathrm{\tau \nu D}\nu^{T}. \end{array}$$

We can now iterate this formula in order to obtain the numerical approximation for the second moment of the ETL at any timestep.

The behaviour of the second moment in time as given by the CME, $S(t)=E(x(t)x^{T}(t))$, is [33,40]

(26) $$\begin{array}{cc} \frac{\mathrm{dS}(t)}{\mathrm{dt}}=\mathrm{WS}(t)+S(t)W^{T}+\mu (t)d^{T}\nu^{T}+\nu d\mu^{T}(t) & \;\\ \;+\mathrm{\nu B}(t)\nu^{T}+\mathrm{\nu D}\nu^{T}, \end{array}$$

where *B*(*t*)=diag(*C****μ***(*t*)). By solving Eq. (26) and iterating Eq. (25), we can use the formulation of Eq. (15) to quantify the bias and Monte Carlo errors of the ETL. A similar approach can also be used for the MPTL and TTTL.

## List of abbreviations

ODE;Ordinary differential equation, SDE;Stochastic differential equation, CME;Chemical Master Equation, SSA;Stochastic simulation algorithm, ETL;Euler *τ*-leap, xETL;Extrapolated Euler *τ*-leap, xxETL;Double-extrapolated Euler *τ*-leap, MPTL, xMPTL, xxMPTL; Midpoint *τ*-leap and extrapolated and double-extrapolated versions,TTTL, xTTTL, xxTTTL;Theta-trapezoidal *τ*-leap and extrapolated and double-extrapolated versions, CV;Coefficient of variation

## Competing interests

The authors declare that they have no competing interests.

## Authors’ contributions

The main ideas were jointly discussed and developed by all authors, and all authors wrote the manuscript. The global error expansion was mainly calculated by KCZ. TSz performed the simulations and analysed them. All authors have read and approved the final version of the manuscript.
